# COVID‐19 in cancer patients on active systemic therapy – Outcomes from LMIC scenario with an emphasis on need for active treatment

**DOI:** 10.1002/cam4.3423

**Published:** 2020-10-31

**Authors:** Anant Ramaswamy, Lingaraj Nayak, Nirmalya Roy Moulik, Manju Sengar, Girish Chinnaswamy, Kunal Jobanputra, Minit J. Shah, Akhil Kapoor, Amit Joshi, Amit Kumar, Anant Gokarn, Avinash Bonda, Badira Cheriyalinkal Parambil, Maya Prasad, Bhausaheb Bagal, Chetan Dhamne, Gaurav Narula, Hasmukh Jain, Jaya Ghosh, Jayashree Thorat, Jyoti Bajpai, Nandini Menon, Navin Khattry, Prabhat Bhargava, Sachin Punatar, Seema Gulia, Shripad Banavali, Sudeep Gupta, Sujay Srinivas, Sushmita Rath, Tushar Vora, Vanita Noronha, Vijay M. Patil, Vikas Ostwal, Kumar Prabhash

**Affiliations:** ^1^ Department of Medical Oncology Tata Memorial Hospital Homi Bhabha National Institute (HBNI) Mumbai India

**Keywords:** cancer, COVID‐19, LMICs, RT‐PCR negativity, systemic therapy

## Abstract

**Background:**

There is limited data on outcomes in cancer patients with coronavirus disease 2019 (COVID‐19) from lower middle‐income countries (LMICs).

**Patients and Methods:**

This was an observational study, conducted between 12 April and 10 June 2020 at Tata Memorial centre, Mumbai, in cancer patients undergoing systemic therapy with laboratory confirmed COVID‐19. The objectives were to evaluate cumulative 30‐day all‐cause mortality, COVID‐19 attributable mortality, factors predicting mortality, and time to viral negativity after initial diagnosis.

**Results:**

Of the 24 660 footfalls and 7043 patients evaluated, 230 patients on active systemic therapy with a median age of 42 (1‐75) years were included. COVID‐19 infection severity, as per WHO criteria, was mild, moderate, and severe in 195 (85%), 11 (5%), and 24 (11%) patients, respectively. Twenty‐three patients (10%) expired during follow‐up, with COVID‐19 attributable mortality seen in 15 patients (6.5%). There were no mortalities in the pediatric cohort of 31 (14%) patients. Advanced stage cancer being treated with palliative intent vs others [30‐day mortality 24%% vs 5%, odds ratio (OR) 5.6, 95% CI 2.28‐13.78, *P* < .001], uncontrolled cancer status vs controlled cancer (30‐day mortality37.5%% vs 4%%, OR 14, 95% CI 4.46‐44.16, *P* < .001) and severe COVID‐19 vs mild COVID‐19 (30‐day mortality 71% vs 3%, OR 92.29, 95% CI 26.43‐322.21, *P* < .001) were significantly associated with mortality. The median time to SARS‐CoV‐2 RT‐PCR negativity was 17 days [interquartile range (IQR)17‐28) in the cohort.

**Conclusions:**

The mortality rates in cancer patients with COVID‐19 who are receiving systemic anti‐cancer therapy in LMICSs are marginally higher than that reported in unselected COVID‐19 cohorts with prolonged time to viral negativity in a substantial number of patients. The pediatric cancer patients tended to have favorable outcomes.

## Graphical abstract

COVID‐19 attributable mortality in cancer patients on systemic therapy in LMICs appears lower than published data and slightly more than an unselected patient's cohort. Delayed recovery in terms of SARS COV‐2 negativity is seen in these patients. Treating Cancer remains the priority.

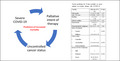

## 1 INTRODUCTION

The COVID‐19 pandemic has impacted the entire globe with different countries in different stages of the pandemic. The incidence in India is still on a gradually rising trend, though case fatality rates (approximately 3%) appear lower than available data from European countries and North America.^1^

Patients with active cancers undergoing systemic treatment like chemotherapy, are presumed to be at higher risk for COVID‐19 as well as COVID‐19‐related complications. Large prospective datasets from CCC‐19 consortium as well as the UKCCMP group have now confirmed the increased mortality rates in cancer patients as compared to the general population.^2^, ^3^ Such data are paramount in determining risk‐benefit analysis for patients when being planned for cancer‐directed therapy. However, there is limited data to differentiate the behavior and outcomes inpatients across stages and disease status receiving systemic treatments of varying intensities. Differences between solid and hematolymphoid malignancies as well as controlled and uncontrolled disease states are expected, though not unequivocally described. Outcomes of pediatric patients on active systemic treatment has also not been widely reported.^4^

Besides definitive end‐points like intensive care unit (ICU) admission and case fatality rates, a further point of interest is the potential delay in recovery from COVID‐19 in cancer patients in terms conversion from RT‐PCR positivity to negativity.^5^, ^6^ In addition to being a relevant clinical endpoint, recovery is an important aspect of the pandemic in terms of requirements for home isolation, ward admissions, duration of stay in a hospital environment, and resumption of anti‐cancer therapy.

A COVID Action group (CAG) was formed in anticipation of patients with cancer and COVID‐19 being treated in our institution.^7^, ^8^ The current study prospectively started collecting data of COVID‐19 positive patients with cancer being treated with systemic therapy at Tata Memorial centre, Mumbai, from 12 April 2020. We aimed to describe clinical and demographic characteristics in this cohort of patients as well as attempted to correlate interactions between cancer status, cancer treatment, and COVID‐19‐related outcomes.

## 2 METHODS

### 2.1 Patients

A prospective database of patients with COVID‐19 was maintained in the Department of Medical Oncology at Tata Memorial centre, Mumbai. Institutional ethical clearance for the maintenance and continuation of the database was applied for and obtained on 18 June 2020. The study has also been submitted for Clinical Trials Registry of India (CTRI) registration; REF/2020/07/035004.

Patients seen in the department with active cancer and diagnosed with COVID‐19 were included into the study. Patients were tested as per institutional policy. As per institution protocol and international standards, COVID‐19 was diagnosed if an RT‐PCR assay from oropharyngeal or nasopharyngeal swab was positive for SARS‐CoV‐2. Repeat testing was conducted at 14 days post initial RT‐PCR positivity for evaluation of recovery. Another repeat test 24 hours later was conducted to confirm recovery. If RT‐PCR positivity persisted, then repeat testing was conducted every 3‐4 days till RT‐PCR negativity. Patients on active chemotherapy or those who had received systemic therapy within the past 12 months were included in the study. Patients whose immediate prior event were surgery/radiotherapy or were being planned for surgery/radiotherapy as the initial cancer directed treatment were not included in this study. Patients receiving concurrent chemoradiation were eligible for inclusion into study.

Current cancer status was defined as controlled cancer status, which included patients with solid tumors undergoing perioperative treatment, advanced/metastatic solid tumor patients with evidence of at least stable disease within 3 months prior and patients in remission on follow up; uncontrolled cancer status, which included patients on active symptom control (ASC) and patients with advanced disease with no evidence of response on more recent evaluation; active treatment, which included patients on active systemic therapy, but without any response evaluation suggestive of disease control or otherwise. Systemic treatment was divided into myelosuppressive and nonmyelosuppressive (predominantly targeted therapy like tyrosine kinase inhibitors). All patients underwent treatment as per decisions by individual treating physician's disease management groups. Treatment records and outcomes were monitored till 25 June 2020.

### 2.2 Data collection and entry

Data collection and entry were done by medical oncologists who were members of the CAG, with additional inputs on treatment modalities obtained from individual treating groups and physicians. Patient demographics, details of cancer and treatment, and the course of the COVID‐19 illness were entered into SPSS software, version 23. The severity of COVID‐19 was categorized as per WHO guidelines into mild, moderate, and severe.

### 2.3 Endpoints

The primary endpoint of the study was defined as 30‐day mortality, irrespective of cause during the course of COVID‐19. This was calculated from the date of diagnosis of COVID‐19 by RT‐PCR to date of death, if it occurred within 30 days of diagnosis of COVID‐19. COVID‐19‐related mortality was also reported when the investigators adjudged mortality as caused due to COVID‐19 alone as opposed to uncontrolled cancer and COVID‐19 infection.

The secondary endpoint was assessment of the median duration taken for conversion from COVID‐19 RT‐PCR positivity to negativity. A negative COVID‐19 RT‐PCR result under a cut‐off of 14 days was considered as normal, while any period beyond 14 days for detection of RT‐PCR negativity was considered as delayed.

### 2.4 Statistical analysis

Analysis of primary endpoint with respect to various factors predicting for mortality was performed using logistic regression analysis. The factors evaluated were male gender, age >60 years, history of smoking, presence of diabetes mellitus, presence of hypertension, hematolymphoid vs solid tumor malignancies, intent of treatment (curative vs palliative), cancer status and severity of COVID‐19 infection. Factors influencing delayed recovery from COVID‐19 based on RT‐PCR negativity were evaluated by univariate analysis. Data processing and visualization were performed using SPSS for Windows software, version 23.0 (SPSS, Chicago, IL).

## 3 RESULTS

### 3.1 Baseline features and correlation with mortality

There were 24 660 footfalls and 7043 patients evaluated in the Dept. of Medical Oncology in the period from 23 March 2020 to 10 June 2020. Among these patients, 230 patients with active cancer on systemic therapy who had documented SARS‐CoV‐2 infection were eligible for analysis in the study. Median follow‐up for the entire cohort was 39 days (range: 36.7‐41.2). Demographic‐, clinical‐, and cancer‐related characteristics for study cohort are in Table 1. Median age was 42 years (range: 1‐75) and 126 (54%) were male. The most prevalent malignancies were acute leukemia in 47 (20%) and gastrointestinal malignancies in 39 (17%) patients. One hundred twenty‐three (53%) patients had evidence of remission or controlled cancer status, while 32 (14%) patients had uncontrolled cancer status or were on active symptom control. Detailed descriptions of cancer subtypes and systemic treatments are given in Tables S1 and S2.

**TABLE 1:** Baseline clinical characteristics of patient cohort

Characteristic	All patients	Patients who died
Gender		
Female	106 (46)	13 (57)
Male	124 (54)	10 (43)
Age (median) (years)	42 (1‐75)	54 (32‐68)
<60	199 (86)	
≥60	31 (14)	5 (22)
Age (years)		
≤12	31 (14)	0
>12	199 (86)	23 (100)
Smoking		
Current smokers	22 (10)	0
Never smokers	196 (85)	21 (91)
Status not known	12 (5)	2 (9)
Comorbidities		
Diabetes mellitus	30 (13)	5 (22)
Hypertension	25 (11)	13
Cardiac illness	2 (1)	0
Primary cancer site		
Hematolymphoid	90 (38)	8 (35)
Lymphoma	31 (13)	5 (22)
Leukemia	47 (20)	2 (9)
Other hematological	12 (5)	1 (4)
Solid tumor malignancies	140 (62)	15 (65)
Gastrointestinal	39 (17)	4 (17)
Breast	30 (13)	3 (13)
Sarcomas	16 (8)	1 (4)
Head and neck	17 (7)	3 (13)
Thoracic	12 (5)	2 (9)
Gynecological	13 (6)	2 (9)
Urological		0
Central nervous system	7 (3)	0
Cancer stage		
Advanced/Metastatic solid malignancies	52 (22)	9 (39)
Stage I‐III (Localized/loco‐regionally advanced) solid malignancies	93 (41)	9 (39)
Hematolymphoid malignancies	85 (37)	5 (22)
Broad treatment intent		
Curative	171 (75)	9 (39)
Palliative	59 (25)	14 (61)
Cancer status		
Controlled cancer	122 (53)	5 (22)
Uncontrolled cancer	32 (14)	12 (52)
No response assessment (On treatment)	76 (33)	6 (26)
Severity of COVID‐19		
Mild	195 (85)	5 (22)
Moderate	11 (5)	1 (4)
Severe	24 (11)	17 (74)
Intensive care admission	8	5 (22)

Note

Data are median or n (%).

The most common presenting symptoms of COVID −19 were fever [145 (63%)], cough [28 (12%)], dyspnea [24 (10%)], myalgia [11 (5%)], and headache [2 (1%)]. COVID‐19 severity was mild in 195 (85%), moderate in 11 (5%), and severe in 24 (10%) patients, respectively. Eight patients (3%) were shifted to the intensive care unit (ICU), of whom 3 patients recovered and were discharged, while 5 patients died. Details of treatment offered to these patients for the management of COVID‐19 are briefly outlined in Table S3.

As of 25 June 2020, 23 patients had died, all within 30 days of COVID‐19 diagnosis. The death was principally attributable to COVID‐19 in 15 (65%), while the remaining eight (35%) patients died likely due to uncontrolled cancers status or treatment related complications with COVID‐19 infection being a concurrent illness. The crude COVID‐19‐related mortality in the cohort of patients was calculated to be 6.5% (15/230). Compared with the rest of the cancer cohort, patients who died had advanced cancers being treated with palliative intent (14/59[24%] vs 9/171[5%]; Odds ratio‐ 5.6 [95% CI: 2.28‐13.78]; *P* < .001), uncontrolled cancer status (12/32[38%] vs 5/122 [4%] {controlled cancer status} vs 6/76 [8%]{on active treatment}):Odds ratio‐ 14[95% CI: 4.46‐44.16]; *P* < .001) and severe COVID‐19 infection status (17/24[71%] vs 1/11 [9%] {moderate COVID‐19} vs 5/195 [3%]{mild COVID‐19}):Odds ratio‐ 92.29 [95% CI: 26.43‐322.21]; *P* < .001). Elderly age (>60 years), history of smoking, presence of diabetes mellitus or underlying hematolymphoid malignancy (as opposed to solid tumor malignancy) did not predict for increased mortality (Table 2).

**TABLE 2:** Logistic regression analysis of factors predicting for 30 days mortality

Variable	Odd ratio (95% CI)	*P* value
Male gender	1.12 (0.47‐2.68)	0.79
Age > 60 years	1.9 (0.66‐5.65)	0.21
Smoking	1.12 (1.07‐1.18)	0.14
Diabetes mellitus	2.02 (0.69‐5.91)	0.20
Hypertension	1.26 (0.35‐4.59)	0.72
Hematolymphoid malignancy	0.81 (0.33‐2.0)	0.82
Intent of treatment		
Curative	1	<0.001
Palliative	5.6 (2.28‐13.78	
Cancer status		
Controlled	1	<0.001
Uncontrolled	14 (4.46‐44.16)	
Active treatment	2.0 (0.59‐6.81)	
Severe COVID‐19 infection		
Mild	1	<0.001
Moderate		
Severe	92.29 (26.43‐322.21)	

### 3.2 Baseline features and correlation with conversion to RT‐PCR negativity

One hundred and seventy‐two patients had reported RT‐PCR negativity at the time of analysis. The remaining 58 (33%) patients included 23 dead patients, and 25 patients who were still on treatment for COVID‐19 infection. Reports were not available from the database for the remaining 10 patients. The median time to SARS‐CoV‐2 seroconversion was 17 days [Interquartile range (IQR): 17‐28] in the evaluable 154 patients. 52 patients (30%; n = 182) tested negative within or at 14 days of initial COVID‐19 diagnosis by RT‐RCR, 52 patients (30%) tested negative between 15 and 21 days, while 50 patients (29%) required beyond 21 days for RT‐PCR negativity. None of the factors evaluated for association with delayed RT‐PCR conversion were found to be significant. The lack of adequate data in patients with severe COVID‐19 infections precluded analysis in this cohort of patients (Table 3).

**TABLE 3:** Logistic regression analysis of factors affecting delayed conversion to RT‐PCR negativity

Variable	Univariate analysis	
	Odd ratio (95% CI)	*P* value
Age (<12 years)	0.5 (0.2‐1.08)	0.15
Age (>60 years)	1.55 (0.2‐1.39)	0.64
Female gender	0.58 (0.36‐1.4)	0.32
Diabetes mellitus	0.58 (0.19‐1.7)	0.33
Hematolymphoid malignancies	1.13 (0.39‐1.9)	0.73
Uncontrolled cancer status	1.3 (0.24‐2.08)	0.5
Nonmyelosuppressive systemic therapy	1.36 (0.22‐1.86	0.41

### 3.3 Special population – pediatric patients (age < 12 years)

Patients in the pediatric age‐group are specially reported due to the limited available data in this age‐group with regard to concurrent cancer and COVID‐19. 31 (14%) patients were aged below 12 years (range: 1‐12) and were available for analysis. 20 patients (65%) patients were on treatment for hematolymphoid malignancies, while the remaining 11 patients (35%) had underlying solid tumor malignancies. Twenty‐three patients (74%) of the pediatric cohort had controlled cancer status, 7 (23%) were on active treatment, while one patient (3%) had uncontrolled cancer status. Thirty‐one patients (97%) presented with mild COVID‐19 infection, while one child presented with severe COVID‐19 infection. There were no mortalities in this cohort.

## 4 DISCUSSION

Health care systems and health care providers across the globe are grappling with the effects and ensuing dramatic changes in functioning because of the COVID‐19 pandemic. There has been a significant shift in resource allocation, medical facilities, and personnel toward dealing with the pandemic at the potential cost of managing other medical conditions.

The current report from the Tata Memorial Centre, Mumbai, a high‐volume standalone cancer centre located in Mumbai, concentrates on the clinical aspects and outcomes of COVID‐19 positive cancer patients on systemic therapy. This report has multiple implications and additions to the emerging literature on cancer patients afflicted with COVID‐19. First, most of the available larger data‐sets on cancer and COVID‐19 has come from North America, Spain, United Kingdom, and China. There is no data from resource constrained, low‐middle‐income countries (LMICs) where simultaneous management of the pandemic and cancer has its own unique set of problems. The current study addresses this aspect comprehensively. Second, the study solely concentrates on a proportion of patients who are likely most vulnerable to infections due to their modified immune status, that is, patients on chemotherapy with a significant cohort of advanced cancers. Third, Mumbai has the highest number of COVID‐19 cases in India and managing cancer patients in this scenario has lessons for other parts of the country and the world where similarly high COVID‐19 incidence rates are seen.^9^ Finally, the outcomes of pediatric patients with cancer and COVID‐19 is encouraging and builds upon the data seen earlier from Memorial Sloane Kettering Cancer Center (MSKCC).

In total, 230 patients who were on active systemic therapy were evaluated in this study. The most prevalent cancers were acute leukemia (20%) and gastrointestinal cancers (17%), which is in contrast to the data from the CCC19 (most prevalent cancers—breast cancer and prostate cancer), but similar to that from the UKCCMP project (most common cancers—hematological and digestive organ malignancies).^2^, ^3^ 22% of patients had advanced cancers, while 25% of patients were being treated with palliative intent therapy. We adopted the broad terminology of “palliative” and “curative” intent treatment as a potential surrogate for cancer stage as well to accommodate for the heterogeneity in cancers being evaluated, though the limitations of this approach are well‐known 3.5% of patients required intensive care unit (ICU) admissions and this is marginally higher compared to country‐wide rates (2.25% as of 29 May 2020) as per available data. However, these ICU admission rates might not be truly reflective of actual ICU requirements for concomitant severe COVID‐19 and cancer due to the nature of hospital policies and reluctance of critical care physicians in using ICU resources for patients with advanced cancers.

Several important conclusions can be drawn from this initial analysis. First, the COVID‐19‐related case fatality rate in the cohort of cancer patients in this study was 6.5%, while the overall 30‐day mortality rate was 10%. The crude COVID‐19‐related case fatality rate has to be evaluated from multiple perspectives. The current study cohort is probably on the higher end of the vulnerability spectrum when compared to all patients with cancer as these patients were majorly on active systemic therapy, which results in an immune system prone to infections and related sequelae. Additionally, some of these patients with advanced cancers have a narrow window for treatment to control disease status. Certain intensive chemotherapeutic regimens entail mortality rates of up to 5% due to treatment‐related complications.^10^ When we combine these salient points with the fact that the current COVID‐19 related case fatality rates in an unselected population in India are pegged at approximately 2.9% (as of 3 July 2020), the scenario appears clearer. Patients with cancer on active systemic therapy are possibly at a slightly higher risk of COVID‐19‐related mortality than the general population but there is a higher probability of cancer‐related mortality if cancer remains untreated; appropriate treatment of these patients in the COVID‐19 pandemic is essential.

The 30‐day case fatality rates in this scenario (10%) appear similar to data from Mount Sinai hospital (death rate—11%), MSKCC (death rate—12%), and the CCC19 (death rate—13%) experience, but markedly lesser than mortality rates seen from the UKCCMP database (28%) and another hospital from New York city (28%).^11^, ^12^ A closer look at the data reveals several possibilities that explain these rates. A majority of patients in this study were classified as having mild COVID‐19 infection (85%) as compared to only 52% in UKCCMP database. Second, our initial institution policy aimed at hospital admission for nearly all patients diagnosed with COVID‐19. This was based on the initial lack of knowledge as well as the uncertainty as to how the course of the COVID‐19 infection would pan out in cancer patients, the logistic constraints a majority of patients faced with regard to lack of adequate home isolation facilities as well as an inability of patients to deal with the limitations of the country‐wide lockdown that was enforced to curb the spread of COVID‐19. Finally, the current study cohort included 25% patients being treated with palliative intent (UKCCMP—43%) and only 14% patients with uncontrolled cancer status (CCC19—25%). These factors and contrasts with available large datasets are significant for purposes of comparison when we look at the subgroups of patients with cancer who appear to be at increased risk for adverse outcomes. In our study, the presence of severe COVID‐19 infection, palliative intent of therapy (as a possible surrogate of advanced cancers) and uncontrolled cancer status appear to be associated with an increased risk of mortality from COVID‐19 in patients with cancer. The significance of these factors appears to have biological rationale in the context of cancer and have been identified in the other data‐sets as well, though not unequivocally. The implications of these findings are multifold. Increased testing for COVID‐19 to pick up patients with minimally symptomatic or asymptomatic COVID‐19 infection should be a priority so that COVID‐19‐related complications are minimized. The treatment of cancer even in the COVID‐19 era should be continued as per existing standards to ensure disease control. Early re‐initiation of therapy with the possibility of continuing cancer directed therapy even during mild COVID‐19 should be considered in the coming times as uncontrolled cancer status is a risk factor for COVID‐19‐related mortality. The concerns over cancer related mortality should possibly override the concern for COVID‐19 and related complications and result in adequate and appropriate treatment of cancer in these patients. At the opposite end of the spectrum, patients with exhausted or limited treatment options should be considered for early palliative care and limited hospital visits to ensure limited exposure to COVID‐19. These findings are fraught with ethical and oncological implications that need detailed discussion within the oncology community as well as within patients and support groups.

The diagnosis of a COVID‐19 infection and survival rates are not the only outcomes that bear evaluation from a health planning point of view. The current study suggests that patients with cancer on active systemic therapy take a median of 17 days to undergo seroconversion to negativity and this longer than in an unselected patient cohort. Only 30% tested patients seroconverted to negativity within or at a 14‐day interval. This is an important factor when cancer patients are counseled with regard to their possible course when afflicted with COVID‐19. Larger data‐sets may verify these findings, which are of practical and epidemiological importance. Severity of COVID‐19 illness also correlates with delayed recovery, but the small number of repeat samples in patients with severe COVID‐19 in our study precludes this conclusion.

It is reassuring to note the outcomes in pediatric cancer patients with COVID‐19. Only one patient was diagnosed with severe COVID‐19 in the study and there were no fatalities in the entire group. The outcomes in this group closely mirror those seen in the much larger study on pediatric patients from the Memorial Sloan Kettering Cancer Center group.^4^

The current study has some limitations, such that some observations need to be interpreted with caution. The diagnosis of COVID‐19 as well as recovery relies on testing with RT‐PCR, which has a well‐known false‐negative rate. This might result in under‐diagnosing COVID‐19 in cancer patients, especially in patients who have mild symptoms. Additionally, the comments on recovery time noted in the study may suffer from the same bias. The study has also evaluated only patients who presented to the hospital for testing and treatment; it does not provide information on patients with possibly advanced cancers who may have ceased treatment and yet developed COVID‐19 and related sequelae in the community. This prevents us from assessing the actual prevalence of COVID‐19 in cancer patients. A selection bias in terms of testing patients with symptoms as opposed to universal testing might result in a further under‐reporting of COVID‐19 prevalence in cancer patients. Conversely, by not commenting of the interactions between COVID‐19 and other components of cancer care like surgery, radiotherapy, and immunotherapy, the noted mortality rates might be an over‐estimation. We have not provided details on biochemical and radiographic findings that would have further helped describing and categorizing these patients. We have briefly enumerated treatment options given to these patients; this is of importance as drugs such as steroids and tocilizumab used in the general population have a different connotation when being considered for use in patients with cancer.

In summary, the data from this report offer a snapshot of the impact of COVID‐19 in cancer patients on active systemic therapy in a high‐volume stand‐alone cancer centre in India. COVID‐19 infection with cancer is not uncommon. Mortality rates in this cohort appears marginally more than an unselected patient cohort, but active disease control via appropriate treatment is of paramount importance to prevent cancer and concurrent COVID‐19‐related mortality. Conversion to COVID‐19 RT‐PCR negativity appears to be delayed in patients with cancer on active systemic therapy. We need to develop strategies to actively treat patients with cancer and COVID‐19 to avoid cancer‐related mortality.

## ETHICAL CLEARANCE

IEC number ‐ IEC/0620/3480/001.

## CONFLICT OF INTEREST

None.

## AUTHOR CONTRIBUTIONS

All authors contributed to conceptualization, data curation, formal analysis, funding acquisition, investigation, methodology, project administration, resources, software, supervision, validation, visualization, writing—original draft, and writing—review and editing.

## Supporting information

Table S1‐S3

## Data Availability

The data that support the findings of this study are available on request from the corresponding author. The data are not publicly available due to privacy or ethical restrictions.
