# Supplementary material for: COVID‐19 in cancer patients on active systemic therapy – Outcomes from LMIC scenario with an emphasis on need for active treatment
Source: Cancer Med. 2020 Oct 31;9(23):8747–53. doi: 10.1002/cam4.3423 (PMC7724305; doi:10.1002/cam4.3423)
Supplement: Supplementary file 1 — Table S1‐S3 [file CAM4-9-8747-s001.docx]

Supplementary table 1 - Individual cancer types and their proportions in cohort

| Individual cancer type | All patients |
| --- | --- |
| Hematolymphoid(age >12 years)   - Lymphoma - Leukemia - Multiple myeloma   Solid tumor malignancies (age > 12 years)   - H & N - Breast - NSCLC - Esophageal (SCC) - Gastro-esophageal Junction and gastric - Biliary tract - Hepatocellular carcinoma - Pancreatic/periampullary - Colorectal - Urinary bladder - Prostate - Ovarian - Endometrial - Cervical - Sarcoma - Neuro - GCT - Melanoma   Pediatric   - Lymphoma - Leukemia - Solid tumor malignancies | 30  32  6  16  30  6  7  6  16  1  2  10  1  2  4  2  6  12  4  2  1  1  20  11 |

Supplementary table 2 – Individual systemic treatment in patient cohort

| Individual cancer type | All patients | Patients who died |
| --- | --- | --- |
| Chemotherapy >3 agents  Triplet chemotherapy  Doublet chemotherapy  Single agent chemotherapy  Endocrine therapy  Targeted therapy (tyrosine kinase inhibitors etc.)  Concurrent chemoradiation  Completed chemotherapy | 37 (16)  25 (11)  81 (35)  49 (21)  10 (4)  17 (7)  04  07 | 4 (17)  1 (4)  6 (26)  4 (17)  2 (9)  2 (9)  1 (4)  3 (13) |

Supplementary table 3 – Treatment for patients with COVID-19

| Treatment | All patients |
| --- | --- |
| Azithromycin  Methylprednisolone  Anticoagulation  Tocilizumab  Lopinavir-Ritonavir  Interferon alpha | 12 (5)  10 (4)  09 (4)  04 (2)  1 (0.5)  1(0.5) |
